# Diagnostic and Pharmacological Potency of Creatine in Post-Viral Fatigue Syndrome

**DOI:** 10.3390/nu13020503

**Published:** 2021-02-04

**Authors:** Sergej M. Ostojic

**Affiliations:** 1 FSPE Applied Bioenergetics Lab, University of Novi Sad, 21000 Novi Sad, Serbia; sergej.ostojic@chess.edu.rs; 2 Faculty of Health Sciences, University of Pecs, H-7621 Pecs, Hungary

**Keywords:** post-viral fatigue syndrome, chronic fatigue syndrome, creatine, GAA, creatine kinase

## Abstract

Post-viral fatigue syndrome (PVFS) is a widespread chronic neurological disease with no definite etiological factor(s), no actual diagnostic test, and no approved pharmacological treatment, therapy, or cure. Among other features, PVFS could be accompanied by various irregularities in creatine metabolism, perturbing either tissue levels of creatine in the brain, the rates of phosphocreatine resynthesis in the skeletal muscle, or the concentrations of the enzyme creatine kinase in the blood. Furthermore, supplemental creatine and related guanidino compounds appear to impact both patient- and clinician-reported outcomes in syndromes and maladies with chronic fatigue. This paper critically overviews the most common disturbances in creatine metabolism in various PVFS populations, summarizes human trials on dietary creatine and creatine analogs in the syndrome, and discusses new frontiers and open questions for using creatine in a post-COVID-19 world.

## 1. Introduction

Post-viral fatigue syndrome (PVFS) is a medical condition that is categorized among other disorders of the nervous system (Code: 8E49) in the eleventh revision of the International Classification of Diseases [1]. According to the current codification system published by the World Health Organization in 2019, PVFS covers chronic fatigue syndrome (CFS) and benign myalgic encephalomyelitis (ME), puzzling conditions previously designated as individual entities, and now systematized under the PVFS umbrella. Although some CFS/ME cases are not preceded by a viral infection [2], all conditions share clinical features that allow for a mutual medical/scientific exploration. Mainly characterized by a prolonged severe post-exercise malaise, an impairment in various cognitive functions, non-inflammatory myalgia and joint pain, and unrefreshing sleep, PVFS has an unknown cause, no pathognomonic diagnostic criteria, and no approved medical treatment (for a detailed review see [3,4,5]). Various viral infections have often been reported before the first appearance of PVFS, including Epstein–Barr virus, cytomegalovirus, coxsackieviruses, and coronaviruses, and the onset might be sudden or gradual [6]. Besides viruses, many physiological and psychological factors appear to work together to predispose an individual to PVFS and to precipitate and perpetuate the illness [7], making this ailment even more baffling and hard to tackle. Beyond other risk factors, creatine shortfall may be one of the hallmarks of PVFS pathology, with compensating for the lack of creatine perhaps seen as an adjunct management strategy in this mysterious disease [8]. This review paper outlines the irregularities of creatine metabolism in PVFS, summarizes studies on creatine supplementation in PVFS and similar syndromes, and discusses new frontiers of using creatine by emphasizing COVID-19 pandemics and post-COVID-19 convalescence and nutritional care.

## 2. Biomarkers of Creatine Metabolism in Post-Viral Fatigue Syndrome

A pioneering biochemical and muscle study from the late 1980s and early 1990s on patients with PVFS revealed minimal changes in surrogate markers of creatine turnover/muscle physiology. A mildly elevated creatine kinase (CK) and indistinguishable muscle biopsies were found in 96 patients who had suffered from the PVFS, although enterovirus RNA was present in the skeletal muscle of some patients up to 20 years after the onset of disease [9]. Preedy and co-workers [10] reported only minor abnormalities or expected outcomes after a quantitative morphometric analysis of skeletal muscle fibers in 22 patients with PVFS. Mean muscle RNA composition (mg RNA/mg DNA) was reduced by 15% in acute onset PVFS, implying lower muscle protein synthetic potential (but not muscle bulk), while plasma carnosinase and CK levels were within normal ranges. Another study demonstrated mostly regular electromyographic and muscle histopathology studies, and normal plasma CK levels in 35 patients with chronic fatigue [11], yet abnormal fiber density was found in several patients who did not have acute-onset PVFS. Wassif and colleagues [12] confirmed that the conventional biochemical markers (e.g., albumin, liver enzymes, CK, and carnosinase) are insensitive to discriminate patients with CFS and other myopathies, while histological examination revealed relatively mild abnormalities in 3 out of 10 patients with PVFS (e.g., macrophage infiltration and muscle fiber atrophy). An exciting trial found that stress-induced neutrophil mobilization might be disrupted in CFS, with healthy women demonstrating a strong correlation between exercise-induced neutrophilia and plasma CK while this link was not observed in the CFS patients [13].

Studies following those seminal trials brought a somewhat better understanding of tissue metabolism of creatine in PVFS by using magnetic resonance spectroscopy (MRS). Wong and co-workers [14] were arguably the first who evaluated skeletal muscle metabolism in CFS during rest and exercise using ^31^P MRS to reflect minute-to-minute intracellular high-energy phosphate metabolism. The authors found that CFS patients and normal controls have similar skeletal muscle metabolic patterns during and after exercise. However, CFS patients reach exhaustion much more rapidly than normal subjects, at which point they also have relatively reduced intracellular concentrations of ATP (adenosine triphosphate), while no intergroup differences were found for muscle phosphocreatine levels. A follow-up study confirmed that no significant metabolic abnormalities are associated with fatigue in CFS patients, although abnormalities may be present in a minority of patients [15]. However, other ^31^P MRS trials reported significantly reduced the maximal rate of post-exercise phosphocreatine resynthesis in CFS patients compared to sedentary controls [16], or decreased resting values of phosphocreatine-to-phosphocreatine plus phosphate and increased pH levels during exercise in the CFS population [17]. This implies CFS-driven perturbation in energy metabolism, although not all studies reported an impaired rate of phosphocreatine resynthesis after exercise [18,19]. Finally, Brooks and colleagues [20] demonstrated a trend of reduced levels of hippocampal creatine in CFS patients as compared with controls (8.6 mM vs. 10.9 mM), suggesting an impaired creatine metabolism in the brain as well.

Chronic fatigue syndrome in childhood also appears to be characterized by vascular and metabolic alterations in the brain [21], with lower blood flow in the temporal and occipital lobes and markedly higher blood flow in the basal ganglia and thalamus in patients with CFS as compared to healthy children. This was accompanied by a notable elevation of the choline-to-creatine ratio in children with CFS, which was arguably the first time that a possible creatine alteration in CFS in the young brain has been described, although no individual levels for brain metabolites were reported. A choline-to-creatine ratio is a well-known surrogate ^1^H MRS biomarker of altered brain metabolism, with elevated levels perhaps demonstrating a reduction in brain creatine (or an elevation in brain choline). Soon afterward, an elevation in the choline-to-creatine ratio in the basal ganglia and white matter was found in patients with histologically mild hepatitis C suffering from CFS [22]. Altered cerebral metabolism was also found in patients with CFS who demonstrated higher *N*-acetyl aspartate-to-choline and choline-to-creatine ratios in the occipital cortex, as compared to healthy controls [23,24]. Various cardiac bioenergetic abnormalities were found in 12 CFS patients, with the mean phosphocreatine-to-ATP ratio in the CFS group tending to be lower than that seen in the control group, with values consistent with significant cardiac impairment [25]. In addition, the half-time for phosphocreatine recovery from end-exercise to baseline levels was prolonged in CFS patients. Van der Schaaf and co-workers [26] used functional brain imaging in 89 women with CFS, evaluating the possible link between the brain metabolism and clinical features of CFS. They found that more pain in CFS was associated with reduced gray matter volume and decreased *N*-acetyl aspartate-to-creatine ratio in the dorsolateral prefrontal cortex. Nevertheless, most of the studies provided no absolute levels of creatine in relevant tissues, making the case for creatine alterations in CFS incomplete.

Widespread metabolic abnormalities in CFS were corroborated in a recent whole-brain magnetic resonance spectroscopy trial [27]. A notably elevated choline-to-creatine ratio was found in the left anterior cingulate, with metabolite ratios correlated with fatigue in seven brain regions. Specifically, creatine levels in the parietal cortex were lower in CFS patients than in the control group (6.4 mM vs. 7.3 mM, *p* = 0.03), while in the putamen, creatine was higher in patients than in controls (6.3 mM vs. 5.7 mM; *p* = 0.01), suggesting location-specific variation in brain creatine in CFS. Patients with CFS also showed higher brain temperatures than healthy controls in several brain regions, suggesting that neuroinflammation, mitochondrial dysfunction, and aberrant neuronal communication may contribute to metabolic perturbations in the CFS brain. An elevated creatine excretion via urine has been identified as a metabolomic signature of fibromyalgia syndrome, a chronic condition similar to PVFS, with creatine urinary loss correlating well with fatigue and pain severity [28]. The increased utilization of creatine to form ATP in CFS has been suggested in a metabolomics trial [29], as illustrated by elevated urinary creatinine (an end product of creatine metabolism) and decreased serum glycine (a precursor of creatine) in blood and urine samples from 34 women with ME/CFS.

In the search for valid diagnostic/prognostic biomarkers of CFS, Nacul and co-workers [30] recently reviewed lab tests from 272 people with CFS and 136 healthy controls participating in the UK ME/CFS Biobank. The authors reported that patients with severe CFS actually have lower CK levels compared to healthy controls and non-severe CFS patients, with differences persisting after adjusting for sex, age, body mass index, muscle mass, disease duration, and activity levels. This interesting discovery was corroborated in a consecutive trial where among the 30 clinical parameters evaluated at the UK ME/CFS Biobank, only blood CK levels showed statistically significant differences between groups, with levels lower in CFS patients than in healthy controls (59.93 U/L vs. 88.67 U/L; *p* = 0.006) [31]. This was accompanied by a CFS-driven dysregulation of microRNA profiles, which represent genes interconnected with neuronal and endocrine-metabolic system pathways, including an upregulation of *NTRK1*, which is essential for the development and survival of neurons, and downregulation of *MECP2* and *AGO2* genes, which provide instructions for modifying chromatin and RNA-mediated silencing, respectively. Low CK activity in CFS is perhaps another indicator of an inadequate turnover of a key enzyme involved in creatine utilization and may be a symptom of the low availability of cellular energy that might involve both mitochondrial and cytosolic pathways [32,33].

## 3. Dietary Creatine and Alternatives in Syndromes with Prolonged Fatigue

Keeping in mind the fact that supplemental creatine has been investigated in a plethora of neurological, neuromuscular, and immune disorders characterized by creatine deficit or perturbation, it is odd that PVFS mainly remained outside of the scope of the creatine research community. A single clinical trial on creatine supplementation in CFS was registered at ClinicalTrials.gov in 2015 (NCT02374112), and the study is still on going, with no results published so far. Besides, only a handful of trials evaluated the effects of supplemental creatine and/or other guanidino compounds in the PVFS population or similar disorders with prolonged fatigue of unknown source.

Almost 20 years ago, Brouwers and co-workers [34] assessed the effect of a polynutrient supplement on the fatigue severity, clinical symptoms, and physical activity of patients with CFS. The authors conducted a prospective randomized placebo-controlled, double-blind trial in 53 CFS patients who received a multi-component product specifically developed to have a high antioxidative capacity for ten weeks. The product contained protein, carbohydrates, fat, trace elements, minerals, vitamins, and other components, including creatine (1200 mg per 100 mL). The authors found no significant differences between the placebo and the treated group on any of the outcome measures. Besides other methodological limitations of this study (e.g., no assessment of the nutritional status of CFS patients prior to the treatment, some components of the mixture having potential contrasting effects), the creatine dosage used here appears to be insufficient as compared to traditional supplementation regimens.

A research group from the University of Sao Paolo evaluated the effects of creatine supplementation in fibromyalgia, a condition similar (if not equivalent) to CFS and characterized by widespread musculoskeletal pain accompanied by fatigue, sleep, memory, and mood issues [35]. The authors supplemented creatine (20 g of creatine monohydrate for five days followed by 5 g per day throughout the trial) to 43 fibromyalgia patients in a randomized, double-blind, placebo-controlled, parallel-group design, and monitored muscle performance, cognitive function, sleep, and tissue metabolism at baseline and 16-week follow up. Creatine intervention provoked higher muscle phosphoryl creatine levels when compared with the placebo group, accompanied by greater dynamic and isometric muscular strength. The mental health domain from the 36-item Short-Form Health Survey was also improved following creatine supplementation, along with incidental memory from the Delay Recall Test, while the other markers of cognition, quality of life, or sleep remained unchanged. Another randomized controlled crossover trial evaluated the effects of supplemental guanidinoacetic acid (GAA), a natural precursor of creatine, in 21 mid-age women with CFS [36]. Three months of oral GAA (2.4 g/day) induced a significant elevation of total muscle creatine levels compared with the placebo group (36.3% vs. 2.4%; *p* < 0.01), complemented by a superior rise in quadriceps isometric strength and maximal oxygen uptake. GAA also attenuated several aspects of fatigue, such as activity, motivation, and mental fatigue, and improved both physical and mental domains of health-related quality of life assessed through the 36-item Short-Form Health Survey. However, GAA demonstrated no effects on the main clinical outcomes, such as general fatigue and musculoskeletal soreness at rest and during activity.

An interesting cross-sectional study assessed the dietary habits and food avoidance-behaviors in women with CFS [37]. Although no homogeneous pattern of food habits was established in this trial, CFS patients appear to often avoid many foods rich in creatine (e.g., meat, milk, and dairy products). A connection between dietary intake of creatine and clinical features of CFS has not been established so far; however, low creatine consumption from food sources may play an essential role in the creatine metabolism irregularities associated with CFS, and perhaps calls for creatine compensation through the prescription of creatine-rich foods and/or creatine supplementation. Jenkins and Raymen also reported intakes below the reference values for animal-based nutrients (e.g., vitamin D, vitamin A, calcium, zinc, and iron) in CFS patients [38]. To understand this under-investigated disorder will require careful nutritional approaches.

## 4. Alternative Mechanisms of Creatine Action

**Figure 1:**
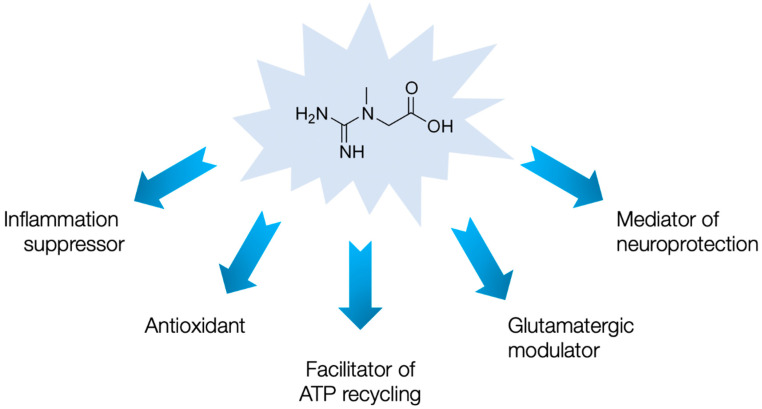
Possible mechanisms of creatine action in post-viral fatigue syndrome.

Both animal studies and human trials indicate the beneficial effects of supplemental creatine in domains beyond cellular bioenergetics, including neuroprotection, immunomodulation, and antioxidant activity (for a detailed review see [39]), domains often compromised in syndromes with chronic fatigue. That being said, creatine may help individuals cope with PVFS through several auxiliary means (Figure 1).

For instance, creatine can act as a mediator of neuroprotection in a variety of neurological conditions, including traumatic brain injury [40], neurodegenerative diseases [41], and cerebral ischemia [42]. This perhaps happens due to a creatine-mediated inhibition of the mitochondrial permeability transition pore (MPTP), an inner membrane protein linked to neuronal cell death, by stabilizing mitochondrial CK and stimulating the production of phosphocreatine, which stabilizes ATP levels in the neuron [43]. Since neuronal mitochondrial dysfunction and the opening of the MPTP play a vital role in PVFS development [44], creatine may renew mitochondrial viability and perhaps alleviate the cognitive dysfunction seen in the syndrome. In addition, creatine can act as a suppressor of acute and chronic inflammation by downregulating membrane proteins (such as toll-like receptors) that play a role in innate immunity [45]. Toll-like receptor upregulation seems to trigger an inflammatory signaling cascade leading to neuroinflammation and neurodegeneration in CFS/ME [46], and supplemental creatine may limit this hyperimmune response. Furthermore, the antioxidant activity of creatine emerges as an additional mechanism that is likely to play a supportive role in the creatine-cytoprotection paradigm [47]. For instance, creatine possesses direct antioxidant activity and can protect mitochondrial DNA from ROS-mediated damage [48]. Owing to the fact that oxidative stress is a major contributor to debilitating chronic fatigue [49], using supplemental creatine to alleviate oxidative damage might be a promising practice in PVFS. Finally, creatine acts as a partial agonist of central GABA_A_ receptors [50], and a modulator of the NMDA receptor [51], both employed in regulating glutamatergic function. An impaired glutamatergic regulation and transport, accompanied by endotoxin intolerance, have been suggested in fatigue syndromes [52], and creatine may play a putative role as a fine-tuning agent of glutamate conveyance in the PVFS brain.

## 5. Open Questions and Creatine in the Post-COVID-19 World

Before considering of whether dietary creatine (and creatine analogs) has any therapeutic value in PVFS, a number of issues have to be addressed in more detail. Firstly, a possible creatine deficiency in the syndrome should be described in terms of demographics that include age, gender, ethnicity, co-morbidities, disease severity and duration, and tissue specificity, among others. Secondly, the intervention protocols should be scrutinized for both prospective and retrospective approaches, with creatine administered being throughout the acute phase of viral infection to induce PVFS, or during an episode of persistent PVFS. We also have to investigate the effects of dietary creatine in tissues besides the skeletal muscle, evaluating brain creatine in PVFS in a region-specific manner, and alternative mechanisms of creatine supplementation via both patient- and clinician-reported outcomes and biomarkers. Creatine intervention specifics in PVFS should also be probed for the chemical formulation that is most effective for sufficient brain uptake, the acceptable dosages in terms of clinical efficacy and pharmacovigilance, and the optimal time span of creatine intervention due to the rather prolonged character of the disease. Specifically, recent data suggest that creatine can cross the blood–brain barrier but only with poor efficiency [53]. The creatine transporter (CT1 or SLC6A8) is not present in the astrocytic feet, which covers ~ 98% of the blood–brain barrier [54], implying somewhat limited uptake of creatine by the brain from the circulation. This problem might be overcome by modifying the creatine molecule to allow it to cross biological membranes by designing lipophilic creatine derivatives that could more adequately enrich the brain creatine pool [55]. Robust research designs that incorporate well-sampled long-term randomized controlled multi-center trials are highly warranted in various PVFS cohorts, including survivors of the coronavirus disease 2019 (COVID-19).

The COVID-19 pandemic caused by the severe acute respiratory syndrome coronavirus 2 (SARS-CoV-2) may bring PVFS into focus since coronaviruses are already recognized for their role in PVFS etiopathogenesis [56]. The medical community has started to see a large number of patients experiencing post-viral persistent fatigue following SARS-CoV-2 infection [57], calling for an effective and affordable treatment for COVID-19 convalescents. Dietary creatine has been recently suggested as a possible adjuvant therapeutic agent for use in COVID-19 recovery [58], due to the beneficial effects demonstrated during rehabilitation in various pulmonary conditions. Being a safe and inexpensive dietary compound, creatine should be investigated post-haste as a possible component of nutritional care for post-COVID-19 fatigue syndrome, along with other nutraceuticals, to reduce post-viral fatigue, promote a swift recovery, and fortify future resistance in often poorly nourished patients [59]. However, like other promising therapeutics for post-COVID-19 subjects, creatine requires accelerated yet attentive research and approval pathways, with sufficient efficacy and safety guarantees. In particular, the effects of creatine on renal function in elderly COVID-19 convalescents should be carefully monitored due to age- and disease-specific kidney impairments that might be exacerbated by creatine intervention.

## 6. Conclusions

Currently, there is not enough evidence to unequivocally endorse supplemental creatine for PVFS. However, the findings from initial trials on the metabolic substrate of PVFS, along with promising results from interventional studies, emphasize the need to explore creatine and similar compounds in this ever-prevalent yet baffling disorder. The need for an effective, low-risk, and affordable dietary intervention to tackle post-COVID-19 fatigue, which is going to remain an issue for years to come, perhaps provides a unique research opportunity to explore creatine in PVFS using expedited yet diligent approaches.

## Data Availability

Not applicable.

## References

[1] World Health Organization (2019). International Classification of Diseases for Mortality and Morbidity Statistics (11th Revision). https://icd.who.int/browse11/l-m/en.

[2] Unger E.R., Lin J.S., Brimmer D.J., Lapp C.W., Komaroff A.L., Nath A., Laird S., Iskander J. (2016). CDC grand rounds: Chronic fatigue syndrome-advancing research and clinical education. MMWR Morb. Mortal. Wkly. Rep..

[3] Lim E.J., Son C.G. (2020). Review of case definitions for myalgic encephalomyelitis/chronic fatigue syndrome (ME/CFS). J. Transl. Med..

[4] Sandler C.X., Lloyd A.R. (2020). Chronic fatigue syndrome: Progress and possibilities. Med. J. Aust..

[5] Kim D.Y., Lee J.S., Park S.Y., Kim S.J., Son C.G. (2020). Systematic review of randomized controlled trials for chronic fatigue syndrome/myalgic encephalomyelitis (CFS/ME). J. Transl. Med..

[6] Morris G., Berk M., Walder K., Maes M. (2016). The putative role of viruses, bacteria, and chronic fungal biotoxin exposure in the genesis of intractable fatigue accompanied by cognitive and physical disability. Mol. Neurobiol..

[7] Afari N., Buchwald D. (2003). Chronic fatigue syndrome: A review. Am. J. Psychiatry.

[8] Ostojic S.M. (2020). Postviral fatigue syndrome and creatine: A piece of the puzzle?. Nutr. Neurosci.

[9] Archard L.C., Bowles N.E., Behan P.O., Bell E.J., Doyle D. (1988). Postviral fatigue syndrome: Persistence of enterovirus RNA in muscle and elevated creatine kinase. J. R. Soc. Med..

[10] Preedy V.R., Smith D.G., Salisbury J.R., Peters T.J. (1993). Biochemical and muscle studies in patients with acute onset post-viral fatigue syndrome. J. Clin. Pathol..

[11] Connolly S., Smith D.G., Doyle D., Fowler C.J. (1993). Chronic fatigue: Electromyographic and neuropathological evaluation. J. Neurol..

[12] Wassif W.S., Sherman D., Salisbury J.R., Peters T.J. (1994). Use of dynamic tests of muscle function and histomorphometry of quadriceps muscle biopsies in the investigation of patients with chronic alcohol misuse and chronic fatigue syndrome. Ann. Clin. Biochem..

[13] Cannon J.G., Angel J.B., Abad L.W., O’Grady J., Lundgren N., Fagioli L., Komaroff A.L. (1998). Hormonal influences on stress-induced neutrophil mobilization in health and chronic fatigue syndrome. J. Clin. Immunol..

[14] Wong R., Lopaschuk G., Zhu G., Walker D., Catellier D., Burton D., Teo K., Collins-Nakai R., Montague T. (1992). Skeletal muscle metabolism in the chronic fatigue syndrome. In vivo assessment by 31P nuclear magnetic resonance spectroscopy. Chest.

[15] Barnes P.R., Taylor D.J., Kemp G.J., Radda G.K. (1993). Skeletal muscle bioenergetics in the chronic fatigue syndrome. J. Neurol. Neurosurg. Psychiatry.

[16] McCully K.K., Natelson B.H., Iotti S., Sisto S., Leigh J.S. (1996). Reduced oxidative muscle metabolism in chronic fatigue syndrome. Muscle Nerve.

[17] Block W., Träber F., Kuhl C.K., Keller E., Lamerichs R., Karitzky J., Rink H., Schild H.H. (1998). 31P-MR- [31P-mr spectroscopy of peripheral skeletal musculature under load: Demonstration of normal energy metabolites compared with metabolic muscle diseases]. Rofo.

[18] McCully K.K., Smith S., Rajaei S., Leigh J.S., Natelson B.H. (2003). Blood flow and muscle metabolism in chronic fatigue syndrome. Clin. Sci..

[19] McCully K.K., Smith S., Rajaei S., Leigh J.S., Natelson B.H. (2004). Muscle metabolism with blood flow restriction in chronic fatigue syndrome. J. Appl. Physiol..

[20] Brooks J.C., Roberts N., Whitehouse G., Majeed T. (2000). Proton magnetic resonance spectroscopy and morphometry of the hippocampus in chronic fatigue syndrome. Br. J. Radiol..

[21] Tomoda A., Miike T., Yamada E., Honda H., Moroi T., Ogawa M., Ohtani Y., Morishita S. (2000). Chronic fatigue syndrome in childhood. Brain Dev..

[22] Forton D.M., Allsop J.M., Main J., Foster G.R., Thomas H.C., Taylor-Robinson S.D. (2001). Evidence for a cerebral effect of the hepatitis C virus. Lancet.

[23] Puri B.K., Counsell S.J., Zaman R., Main J., Collins A.G., Hajnal J.V., Davey N.J. (2002). Relative increase in choline in the occipital cortex in chronic fatigue syndrome. Acta Psychiatr. Scand..

[24] Chaudhuri A., Condon B.R., Gow J.W., Brennan D., Hadley D.M. (2003). Proton magnetic resonance spectroscopy of basal ganglia in chronic fatigue syndrome. Neuroreport.

[25] Hollingsworth K.G., Jones D.E., Taylor R., Blamire A.M., Newton J.L. (2010). Impaired cardiovascular response to standing in chronic fatigue syndrome. Eur. J. Clin. Investig..

[26] Van der Schaaf M.E., De Lange F.P., Schmits I.C., Geurts D.E.M., Roelofs K., van der Meer J.W.M., Toni I., Knoop H. (2017). Prefrontal structure varies as a function of pain symptoms in chronic fatigue syndrome. Biol. Psychiatry.

[27] Mueller C., Lin J.C., Sheriff S., Maudsley A.A., Younger J.W. (2020). Evidence of widespread metabolite abnormalities in Myalgic encephalomyelitis/chronic fatigue syndrome: Assessment with whole-brain magnetic resonance spectroscopy. Brain Imaging Behav..

[28] Malatji B.G., Meyer H., Mason S., Engelke U.F.H., Wevers R.A., van Reenen M., Reinecke C.J. (2017). A diagnostic biomarker profile for fibromyalgia syndrome based on an NMR metabolomics study of selected patients and controls. BMC Neurol..

[29] Armstrong C.W., McGregor N.R., Lewis D.P., Butt H.L., Gooley P.R. (2015). Metabolic profiling reveals anomalous energy metabolism and oxidative stress pathways in chronic fatigue syndrome patients. Metabolomics.

[30] Nacul L., de Barros B., Kingdon C.C., Cliff J.M., Clark T.G., Mudie K., Dockrell H.M., Lacerda E.M. (2019). Evidence of clinical pathology abnormalities in people with myalgic encephalomyelitis/chronic fatigue syndrome (me/cfs) from an analytic cross-sectional study. Diagnostics.

[31] Almenar-Pérez E., Sarría L., Nathanson L., Oltra E. (2020). Assessing diagnostic value of microRNAs from peripheral blood mononuclear cells and extracellular vesicles in myalgic encephalomyelitis/chronic fatigue syndrome. Sci. Rep..

[32] Germain A., Ruppert D., Levine S.M., Hanson M.R. (2017). Metabolic profiling of a myalgic encephalomyelitis/chronic fatigue syndrome discovery cohort reveals disturbances in fatty acid and lipid metabolism. Mol. Biosyst..

[33] Tomas C., Brown A., Strassheim V., Elson J.L., Newton J., Manning P. (2017). Cellular bioenergetics is impaired in patients with chronic fatigue syndrome. PLoS ONE.

[34] Brouwers F.M., Van Der Werf S., Bleijenberg G., Van Der Zee L., Van Der Meer J.W. (2002). The effect of a polynutrient supplement on fatigue and physical activity of patients with chronic fatigue syndrome: A double-blind randomized controlled trial. QJM.

[35] Alves C.R., Santiago B.M., Lima F.R., Otaduy M.C., Calich A.L., Tritto A.C., de Sá Pinto A.L., Roschel H., Leite C.C., Benatti F.B. (2013). Creatine supplementation in fibromyalgia: A randomized, double-blind, placebo-controlled trial. Arthritis Care Res..

[36] Ostojic S.M., Stojanovic M., Drid P., Hoffman J.R., Sekulic D., Zenic N. (2016). Supplementation with guanidinoacetic acid in women with chronic fatigue syndrome. Nutrients.

[37] Trabal J., Leyes P., Fernández-Solá J., Forga M., Fernández-Huerta J. (2012). Patterns of food avoidance in chronic fatigue syndrome: Is there a case for dietary recommendations?. Nutr. Hosp..

[38] Jenkins M., Rayman M. (2005). Nutrient intake is unrelated to nutrient status in patients with chronic fatigue syndrome. J. Nutr. Environ. Med..

[39] Riesberg L.A., Weed S.A., McDonald T.L., Eckerson J.M., Drescher K.M. (2016). Beyond muscles: The untapped potential of creatine. Int. Immunopharmacol..

[40] Dolan E., Gualano B., Rawson E.S. (2019). Beyond muscle: The effects of creatine supplementation on brain creatine, cognitive processing, and traumatic brain injury. Eur. J. Sport Sci..

[41] Marques E.P., Wyse A.T.S. (2019). Creatine as a neuroprotector: An actor that can play many parts. Neurotox. Res..

[42] Balestrino M., Sarocchi M., Adriano E., Spallarossa P. (2016). Potential of creatine or phosphocreatine supplementation in cerebrovascular disease and in ischemic heart disease. Amino Acids.

[43] Beal M.F. (2011). Neuroprotective effects of creatine. Amino Acids.

[44] Morris G., Maes M. (2014). Mitochondrial dysfunctions in myalgic encephalomyelitis/chronic fatigue syndrome explained by activated immuno-inflammatory, oxidative and nitrosative stress pathways. Metab. Brain Dis..

[45] Leland K.M., McDonald T.L., Drescher K.M. (2011). Effect of creatine, creatinine, and creatine ethyl ester on TLR expression in macrophages. Int. Immunopharmacol..

[46] Gambuzza M.E., Salmeri F.M., Soraci L., Soraci G., Sofo V., Marino S., Bramanti P. (2015). The role of toll-like receptors in chronic fatigue syndrome/myalgic encephalomyelitis: A new promising therapeutic approach?. CNS Neurol. Disord. Drug Targets.

[47] Sestili P., Martinelli C., Colombo E., Barbieri E., Potenza L., Sartini S., Fimognari C. (2011). Creatine as an antioxidant. Amino Acids.

[48] Guidi C., Potenza L., Sestili P., Martinelli C., Guescini M., Stocchi L., Zeppa S., Polidori E., Annibalini G., Stocchi V. (2008). Differential effect of creatine on oxidatively-injured mitochondrial and nuclear DNA. Biochim. Biophys. Acta.

[49] Lee J.S., Kim H.G., Lee D.S., Son C.G. (2018). Oxidative stress is a convincing contributor to idiopathic chronic fatigue. Sci. Rep..

[50] Koga Y., Takahashi H., Oikawa D., Tachibana T., Denbow D.M., Furuse M. (2005). Brain creatine functions to attenuate acute stress responses through GABAnergic system in chicks. Neuroscience.

[51] Royes L.F., Fighera M.R., Furian A.F., Oliveira M.S., Fiorenza N.G., Ferreira J., da Silva A.C., Priel M.R., Ueda E.S., Calixto J.B. (2008). Neuromodulatory effect of creatine on extracellular action potentials in rat hippocampus: Role of NMDA receptors. Neurochem. Int..

[52] Rönnbäck L., Hansson E. (2004). On the potential role of glutamate transport in mental fatigue. J. Neuroinflamm..

[53] Béard E., Braissant O. (2010). Synthesis and transport of creatine in the CNS: Importance for cerebral functions. J. Neurochem..

[54] Ainsley Dean P.J., Arikan G., Opitz B., Sterr A. (2017). Potential for use of creatine supplementation following mild traumatic brain injury. Concussion.

[55] Adriano E., Gulino M., Arkel M., Salis A., Damonte G., Liessi N., Millo E., Garbati P., Balestrino M. (2018). Di-acetyl creatine ethyl ester, a new creatine derivative for the possible treatment of creatine transporter deficiency. Neurosci. Lett..

[56] Lam M.H., Wing Y.K., Yu M.W., Leung C.M., Ma R.C., Kong A.P., So W.Y., Fong S.Y., Lam S.P. (2009). Mental morbidities and chronic fatigue in severe acute respiratory syndrome survivors: Long-term follow-up. Arch. Intern. Med..

[57] Townsend L., Dyer A.H., Jones K., Dunne J., Mooney A., Gaffney F., O’Connor L., Leavy D., O’Brien K., Dowds J. (2020). Persistent fatigue following SARS-CoV-2 infection is common and independent of severity of initial infection. PLoS ONE.

[58] Ostojic S.M. (2020). Can creatine help in pulmonary rehabilitation after COVID-19?. Ther. Adv. Respir. Dis..

[59] Butters D., Whitehouse M. (2020). COVID-19 and nutriceutical therapies, especially using zinc to supplement antimicrobials. Inflammopharmacology.

